# Draft Genome Sequence of Streptomyces sp. Strain 150FB, a Mushroom Mycoparasite Antagonist

**DOI:** 10.1128/genomeA.01441-14

**Published:** 2015-04-02

**Authors:** M. T. Tarkka, L. Feldhahn, D. Krüger, N. Arnold, F. Buscot, T. Wubet

**Affiliations:** a Department of Soil Ecology, UFZ-Helmholtz Centre for Environmental Research, Halle, Germany; b German Centre for Integrative Biodiversity Research (iDiv) Halle-Jena-Leipzig, Leipzig, Germany; c Department of Bioorganic Chemistry, Leibniz Institute of Plant Biochemistry, Halle, Germany

## Abstract

Streptomyces sp. strain 150FB, isolated from the cap surface of a bolete mushroom, inhibits the growth of the mycoparasitic *Sepedonium* species. Functional annotation of the strain 150FB draft genome identified 22 putative secondary metabolite biosynthetic gene clusters and genes encoding secreted proteins, which may contribute to the inhibition of the mycoparasite.

## GENOME ANNOUNCEMENT

Mushrooms host a rich community of bacteria and fungi, including both parasites and mutualists. Whereas, for example, bacteria of the genus Pseudomonas cause brown blotch, ginger blotch, and drippy gill diseases on the button mushroom Agaricus bisporus (J. E. Lange) Imbach (1), nitrogen-fixing Bradyrhizobium isolates stimulate Pleurotus mushroom formation (2), and bacteria associated with the white truffle *Tuber borchii* Vittad. contribute to aroma production (3). It has been suggested (4) that the associated bacteria may protect mushrooms from the invasion of parasites. Since mushrooms of *Boletales* s.l. are often infected by molds of the mycoparasitic anamorphic genus *Sepedonium* (teleomorph: Hypomyces; *Ascomycota*) (5), we have isolated yeasts (6) and bacteria (7) from the surface layer of *Boletales* s.l. mushrooms and investigated whether microorganisms on fruiting bodies inhibit this mycoparasite. From the bacterial collection, Streptomyces sp. strain 150FB, isolated from the sterilized outer surface of a *Xerocomus chrysenteron* (Bull.) Quél. mushroom originating from Harzgerode, Germany, efficiently inhibited the growth of the mycoparasite *Sepedonium* sp. The species of Streptomyces commonly express fungal cell wall–active enzymes (8) and produce antifungal substances (9). Our interest on the factors related to the antagonistic capacity of strain 150FB compelled us to define its genetic constitution by sequencing its genome.

Here, we present the draft genome sequence of strain 150FB generated by Illumina HiSeq2000 at Eurofins (Ebersberg, Germany), as well as by 454 Titanium at the Department of Soil Ecology, UFZ, Halle, Germany. After the Illumina sequencing run, the 150FB genome preassembly was performed on 19,232,385 preprocessed single-end reads with a mean size of 81 bp by Velvet version 1.2.07 software (10) (*k*-mer = {21,29,37,45,53,61}). The preassembly included 114,860 contigs with an average size of 509 bp and an *N*_50_ value of 1,263 bp. These contigs were then assembled with 264,494 preprocessed 454 paired-end reads with a mean size of 352 bp by GS *de novo* assembler version 2.7 software (11). The final assembly resulted in 8 scaffolds with an *N*_50_ value of 9,626,015 bp and totaling 9,802,157 bp.

Based on RAST annotation (12), the Streptomyces sp. 150FB genome contains 63 tRNA genes and encodes 8,394 putative proteins, and it has close correspondence to the Streptomyces avermitilis MA-4680 genome. The strain 150FB genome comprises genes potentially involved in the inhibition of fungal growth. These include a set of genes encoding extracellular enzymes involved in the degradation of fungal cell wall polysaccharides (e.g., beta-glucanases, chitin binding proteins, and chitinases), disruption of membranes and proteins (e.g., lipases, serine, and zinc metalloproteases), as well as peroxidases and ribonucleases. Functional annotation by antiSMASH version 2.0 (13) predicted 22 different secondary metabolite gene clusters: the strain 150FB genome harbors 6 nonribosomal peptide synthetases (NRPS), 5 type 1 and 4 type 3 polyketide synthases (PKS), and 3 NRPS type 1 PKS biosynthetic gene clusters. In addition, 2 terpene and 2 siderophore biosynthetic gene clusters were detected. Our work provides an important reference for the identification of factors related to disease suppression on mushrooms and crucial information for future experiments.

### Nucleotide sequence accession number.

The nucleotide sequence has been deposited at GenBank under the accession number JTHL00000000.
